# Differential and Site Specific Impact of B Cells in the Protective Immune Response to *Mycobacterium tuberculosis* in the Mouse

**DOI:** 10.1371/journal.pone.0061681

**Published:** 2013-04-16

**Authors:** Egídio Torrado, Jeffrey J. Fountain, Richard T. Robinson, Cynthia A. Martino, John E. Pearl, Javier Rangel-Moreno, Michael Tighe, Robert Dunn, Andrea M. Cooper

**Affiliations:** 1 Trudeau Institute Inc., Saranac Lake, New York, United States of America; 2 Division of Allergy, Immunology and Rheumatology, Department of Medicine, University of Rochester Medical Center, Rochester, New York, United States of America; 3 Biogen Idec, Cambridge, Massachusetts, United States of America, and San Diego, California, United States of America; Institut Pasteur, France

## Abstract

Cell-mediated immune responses are known to be critical for control of mycobacterial infections whereas the role of B cells and humoral immunity is unclear. B cells can modulate immune responses by secretion of immunoglobulin, production of cytokines and antigen-presentation. To define the impact of B cells in the absence of secreted immunoglobulin, we analyzed the progression of *Mycobacterium tuberculosis* (Mtb) infection in mice that have B cells but which lack secretory immunoglobulin (AID^−/−^µS^−/−^mice). AID^−/−^µS^−/−^ mice accumulated a population of activated B cells in the lungs when infected and were more susceptible to aerosol Mtb when compared to wild type (C57BL/6) mice or indeed mice that totally lack B cells. The enhanced susceptibility of AID^−/−^µS^−/−^ mice was not associated with defective T cell activation or expression of a type 1 immune response. While delivery of normal serum to AID^−/−^µS^−/−^ mice did not reverse susceptibility, susceptibility in the spleen was dependent upon the presence of B cells and susceptibility in the lungs of AID^−/−^µS^−/−^mice was associated with elevated expression of the cytokines IL-6, GM-CSF, IL-10 and molecules made by alternatively activated macrophages. Blocking of IL-10 signaling resulted in reversal of susceptibility in the spleens and lungs of AID^−/−^µS^−/−^ mice. These data support the hypothesis that B cells can modulate immunity to Mtb in an organ specific manner via the modulation of cytokine production and macrophage activation.

## Introduction

Tuberculosis (TB), caused by the intracellular pathogen *Mycobacterium tuberculosis* (Mtb) remains a leading cause of death from a single infectious agent and is estimated to kill approximately 2 million people every year [1]. It is critical that we determine the protective and regulatory components of the immune response to TB in order to improve upon the current vaccine, *Mycobacterium bovis* BCG, or to develop new vaccine strategies [2]. In this respect, our knowledge of the role of B cells during mycobacterial infections remains incomplete, although B cell responses have been shown to promote optimal immunity and immunological memory against intracellular pathogens such as *Chlamydia trachomatis* [3], [4], *Francisella tularensis* [5], *Leishmania major* [6], *Plasmodium chabaudi chabaudi* [7], *Pneumocystis carinii* [8], and *Salmonella enterica* serovar *Typhimurium* [9].

Protective immunity against intracellular pathogens, such as Mtb, is primarily T cell mediated while B cells and antibodies are thought to play a limited role [10]. Indeed, cell-mediated immunity is critical in Mtb, and the IL-12-induced IFN-γ-response provides the primary pathway by which Mtb growth is controlled by the host [11]. B cells have however been shown to play an important role in the regulation of immune responses, both in antibody-dependent and -independent ways [12], [13], [14], [15]. In fact, in addition to producing antibodies, B cells can act as antigen-presenting cells [8], [15], [16], [17] and can influence immune responses by producing cytokines [18], [19], [20].

The role of B cells during TB has been addressed using B cell deficient mice [21], [22], [23], [24], [25]. However, B cell deficient mice may have developmental differences and lack all the functions associated with B cells, making it difficult to discriminate between the specific effector and regulatory functions these cells may be performing during Mtb infection. To date, several studies have shown that low dose aerosol Mtb infection in B cell-deficient mice does not alter lung bacterial burden [21], [22], [23], [24]. Conversely, delivery of a higher aerosol [23] or intravenous [25] dose of Mtb can result in B cell-deficient mice having higher bacterial loads compared to control mice. The inflammatory response can also be impacted as increased T cell and neutrophils were seen in one study [23], while another study reported small and diffuse granulomata when compared to intact mice [21]. In this latter study, transfer of naïve B cells, but not serum, into B cell deficient mice recapitulated the response observed in intact mice [21]. The variable outcomes reported in mice with B cell deficiency may result from altered lymphatic micro-architecture, the dose and route of delivery, the background of the mice, the strain of the bacteria used or possibly the impact of B cell deficiency on the maintenance of gut commensals [26]. In support of this latter point, alteration in the gut commensal bacteria can impact the quality of lung immunity in infection models [27].

Several attempts have been made to investigate the specific role of immunoglobulin during TB and while they have been intriguing they were not fully conclusive. Thus, Mtb-specific IgA antibody can modestly reduce bacterial burden in the lung [28] while a different Mtb-specific antibody could enhance host survival [29] however how these protective effects were achieved is not clear. A role for Fc ligation by circulating immunoglobulin is supported by the fact that ablation of the activating FcγR compromises optimal immunity against Mtb [30], influenza virus [31], *Leishmania* species [6], [32], *Plasmodium berghei* [33], and *S. enterica* [34], [35]. Conversely, deletion of the inhibitory FcγRIIB improves mycobacterial containment and is associated with elevated IFN-γ responses in the lungs [30]. Based on these published observations, defining the extent to which circulating immunoglobulin impacts Mtb infection would appear to be important.

We therefore chose to investigate the function of B cells in mice lacking the ability to secrete immunoglobulin. In these studies we reasoned that B cell functions such as cytokine release and antigen presentation would be present without a confounding impact from circulating immunoglobulin. We therefore infected mice deficient in activation-induced diaminase (AID^−/−^, *Aicda*) and secretory IgM (µS) (*AID* ^−/−^ *µS* ^−/−^). These mice have B cells which are unable to undergo affinity maturation or synthesize secretory antibodies of any isotype and thus while B cells are present, there is no circulating antibody. We were surprised to find that these mice were less able to control Mtb infection than control mice and indeed less able to control infection than mice that lacked B cells. The delivery of exogenous serum did not reverse susceptibility and while these mice exhibited normal T cell activation they had altered phagocyte activation that was dependent upon high levels of IL-10.

## Materials and Methods

### Mice

C57BL/6 and B6.129S2-*Igh-6^tm1Cgn^*/J (µMT) were originally obtained from The Jackson Laboratory (Bar Harbor). B6;129S4-*Igh-6^tm1Che^*/J (µS^−/−^) originally obtained from Dr. R. Corley (Boston University) and Activation-induced cytidine deaminase deficient mice (AID^−/−^) originally obtained from Dr. R. Gerstein (University of Massachusetts Medical School) were previously described [36], [37], [38]. *AID* ^−/−^ and *µS* ^−/−^ mice were intercrossed at the Trudeau Institute in order to generate mice with a normally diverse repertoire of B cells but which were unable to secrete antibodies of any isotype (*AID* ^−/−^ *µS* ^−/−^) [39]. *AID* ^−/−^ *µS* ^−/−^ mice were given Sulfamethoxazole/Trimethoprim (Smx-Tmp) treated food ad libitum up to one week before infection, after which they were given regular mouse food. Some C57BL/6 and *AID* ^−/−^ *µS* ^−/−^ mice were maintained on Smx-Tmp food throughout infection. All mice were bred and maintained at the Trudeau Institute animal facility. Both male and female mice between the ages of 6 to 12 weeks old were used. The level of specific isotype in mouse serum was determined by multiplex bead-based mouse isotype assay kit (Luminex, Millipore) following the manufacturer’s instructions.

### Ethics Statement

All procedures involving live animals were carried out in accordance with the Guide for Care and Use of Laboratory Animals of the National Institutes of Health and individual procedures were approved by the Trudeau Institute Institutional Animal Care and Use Committee.

### Aerosol Infection and Bacterial Load Determination

The H37Rv strain of Mtb was grown in Proskauer Beck medium containing 0.05% Tween 80 to mid-log phase and frozen in 1 ml aliquots at −70°C. For aerosol infections, subject animals were infected using a Glas-Col airborne infection system as previously described in detail [40]. A moderately high dose of approximately 300 colony forming units was used. At day 1 and selected time points post-infection, infected mice were killed by CO_2_ asphyxiation and the organs were aseptically excised. Each of the organs was individually homogenized in saline, followed by plating serial dilutions of the organ homogenate on nutrient 7H11 agar (BD Biosciences). Colony forming units were counted after 3 weeks of incubation at 37°C.

### Antibody and Serum Treatment

For IL-10 signaling blockade, mice were treated i.p. with 350 µg of anti-IL-10R antibody (clone 1B1.3A) or isotype control (clone HRPN) once a week. For B cell depletion, mice were treated i.p. with 250 µg of an anti-mouse CD20 antibody (clone 18B12) once every other week. As a control, mice were treated with the same concentration and isotype of an anti-human CD20 antibody (clone 2B8) [41]. The anti-CD20 antibodies were provided by Biogen Idec, San Diego, CA. Some mice received 200 µls of normal mouse serum delivered intraperitoneally every three days from day 15 to day 60.

### Radiation Bone-marrow Chimeras

Donor bone marrow from 6–8 week old B6 or *AID* ^−/−^ *µS* ^−/−^ mice was harvested via perfusion of the femur and tibia medullary cavities with cold complete DMEM. Marrow suspensions were pelleted and subsequently resuspended in RBC lysis buffer (155 mM NH_4_Cl, 10 mM KHCO_3_) to remove red blood cells; cells were then resuspended at 5×10^7^ cells/mL in sterile saline. Recipient mice received 2×5 Gy (500 rad) doses of whole-body irradiation 3 h apart. Immediately following the second dose, mice were injected i.v. with 200 µl of indicated marrow preparation (i.e. 1×10^7^ total bone marrow cells). Mice were allowed 6–8 weeks to reconstitute before use in experiments. Recipients were kept on antibiotic-containing food 4 weeks after irradiation/bone marrow transfer. Two weeks prior to experimental infection bone marrow recipients were taken off of antibiotic-containing food.

### Lymphocyte Isolation for Flow Cytometry and ELISpot

A single-cell suspension was prepared from the spleen or mediastinal lymph node by passing the organ through a 70-µm nylon cell strainer followed by treatment with RBC lysis buffer. Lung cell suspensions were prepared by perfusing cold saline containing heparin through the heart until the lungs appeared white, whereupon they were removed and sectioned in ice-cold medium. Dissected lung tissue was then incubated in collagenase IX (0.7 mg/ml) and DNase (30 µg/ml) (both from Sigma-Aldrich) at 37°C for 30 min. Digested lungs were disrupted by passage through a 70-µm nylon cell strainer and the resultant single-cell suspension was treated with RBC lysis buffer. Cells prepared in this way were used for ELISpot and flow cytometric analyses.

Cells were stained with conjugated antibodies for 30 minutes on ice. For intracellular cytokine staining, cells were cultured in 50 ng/ml of PMA, 1 µg/ml of Ionomycin and 10 µg/ml of Brefeldin A (all from Sigma-Aldrich) for 4–6 h before being surface stained, fixed and permeabilized using the Cytofix/Cytoperm kit (BD Biosciences) followed by intracellular protein staining for 30 minutes on ice. Antibodies specific for CD8 (clone 53–6.7) and CD19 (clone 1D3) were from BD biosciences; IgM (clone II/41), IgD (clone 11–26), CD4 (clone GK1.5), CD3 (clone 17A2), CD44 (clone IM7), IFN-γ (clone XMG1.2), and TNF (clone MP6-XT22) were from eBioscience. Samples were acquired on a LSRII Special Order System flow cytometer (BD Biosciences). All the data were analyzed using FlowJo software (TreeStar).

Detection of antigen-specific IFN-γ-producing cells from infected organs was conducted using an ELISpot assay as previously described [42]. The frequency of responding cells was determined and applied to the number of cells per sample to generate the total number of responding cells per organ. Cells cultured in the absence of antigen or cells from uninfected mice were used as controls.

### Histology and Immunohistochemistry

The caudal lobe of each lung was inflated with 10% neutral buffered formalin and processed routinely for light microscopy by hematoxylin and eosin stain (Colorado Histo-Prep, Fort Collins, CO). Sections were screened and scored in a blinded manner by a qualified pathologist at Colorado Histo-Prep (0: absent; 1: minimal; 2: mild; 3: moderate; 4: marked).

For immune-fluorescence staining, tissue sections were deparaffinized with xylene and rehydrated in steps from absolute ethanol to distilled water. Antigens were retrieved using the Citrate buffer pH6.0 (Thermo Fisher cat# AP-9003-500) at 95°C for 30 minutes. Tissues were blocked with 5% (v/v) normal goat serum in PBS containing 0.1% Tween 20. Sections were first probed overnight with a IgM rat anti-mouse IL-10 (Santa Cruz biotechnology Cat# sc-73309) followed by a biotin conjugated anti-rat Ig (Pharmingen Cat# 559286). Finally, sections were probed with streptavidin conjugated with Alexafluor 647 (Invitrogen Cat# S21374) and phycoerythrin conjugated Rat anti-mouse B220 (clone RA3-6B2 from BD Pharmingen Cat# 553090). Hoechst was used to counterstain and detect nuclei. Images were obtained with a Leica SP5 confocal microscope (Leica Microsystems, Germany) and the light emissions detected using the appropriate bandwidth settings and separate PMT’s. The data was collected as Leica image files (LIF) using LAS-AF version 2.2.1 software (Leica) and converted into TIF files using Fiji software (http://fiji.sc/wiki/index.php/Fiji).

### Real Time RT-PCR and Lung Cytokine Quantification

RNA was extracted from total lung tissue and analyzed by real-time PCR as previously described [42]. Quantification of protein in lung tissue was determined using the upper right lobe of infected mice after homogenization in Tissue Extraction Reagent I (Invitrogen), containing a Protease Inhibitor cocktail (Sigma-Aldrich) following the manufacturer’s instructions. Samples were spun at 25,000 g for 5 minutes and supernatants were filtered and frozen at −80°C. Supernatants were analyzed using a multiplex bead-based mouse cytokine assay kit (Luminex, Millipore) following the manufacturer’s instructions. The same protein preparations were analyzed for arginase activity using the QuantiChrom Arginase Assay Kit (Bioassay systems, CA) according to the manufacturer’s instructions.

### Statistical Analysis

CFU data were Log_10_ transformed before analysis. GraphPad Prism Software was used to perform Student’s *t* tests or one-way ANOVA with Tukey’s multiple comparisons post-test. A *P* value ≤0.05 was considered significant.

## Results

### Mice with B Cells Unable to Secrete Antibodies (AID^−/−^µS^−/−^) are More Susceptible to Mtb Infection than Intact Mice

**Figure 1:**
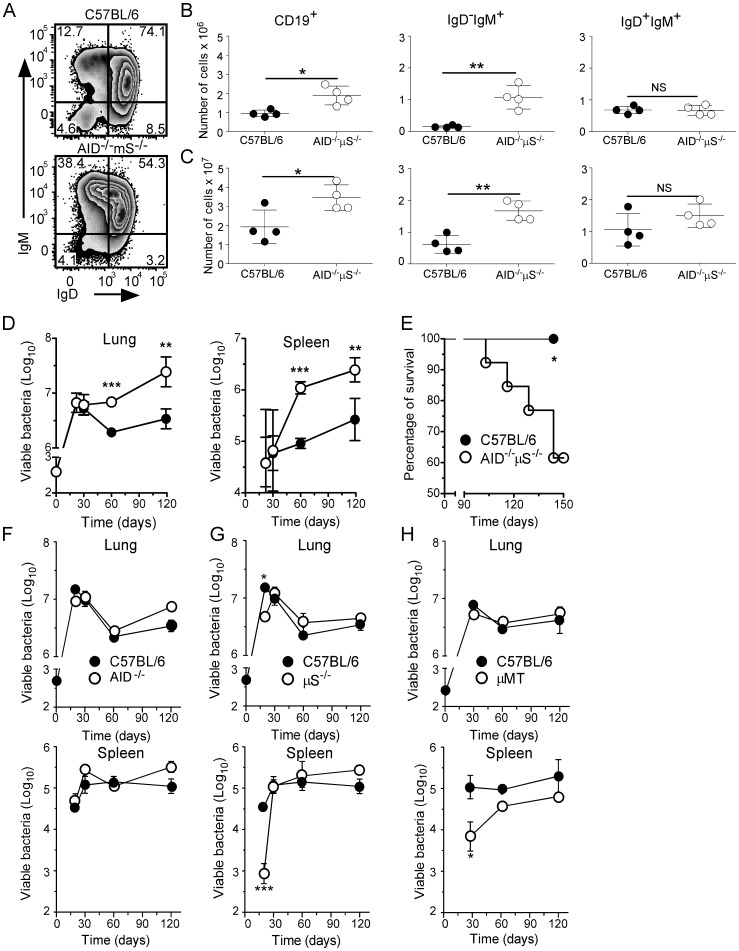
AID^−/−^µS^−/−^ mice have an altered B cell population in the lung and are more susceptible to aerosol infection with Mtb. C57BL/6 (filled circles) and AID^−/−^µS^−/−^ mice (opened circles) were infected with Mtb H37Rv via the aerosol route. (A–C) At day 30 of infection, B cells (gated on live lymphocytes and CD19 expression) were analyzed for the expression of IgM and IgD by flow cytometry. The frequency (A) and total number of CD19^+^ cells, CD19^+^IgD^−^IgM^+^ cells and CD19^+^IgD^+^IgM^+^ cells in the lung (B) and spleen (C) was calculated. (D) The bacterial burden was determined in lungs and spleen over time (*n* = 4). (E) Survival of infected mice was determined over the course of the experimental infection (*n* = 5). (A–E) One experiment representative of at least three independent experiments is shown *, p<0.05**, p<0.01; ***, *p*<0.001 by Student’s *t* test. (F–H) C57BL/6 (filled circles), AID*^−/−^* (F), µS^−/−^ (G) and µMT (H) mice (opened circles) were infected, and the bacterial burden was determined in the lungs and spleen over time (*n* = 4–8). Data sets from a total of two experiments were combined. *, p<0.05; ***, p<0.001 by Student’s *t* test.

To define the role of B cell effector functions other than antibody secretion during TB, we compared the progression of Mtb infection between C57BL/6 mice and mice with a B cell compartment, but which are unable to secrete antibodies (AID^−/−^µS^−/−^ mice). We first assessed the impact of the gene deletions on the B cell populations in the lungs of infected mice using flow cytometry to characterize CD19^+^ cells (B cells) for IgM and IgD expression. We found that AID^−/−^µS^−/−^ mice had higher numbers of CD19^+^ cells in their lungs (Figure 1B) and spleens (Figure 1C) and a significantly higher frequency and number of activated CD19^+^IgD^−^IgM^+^ cells in both the lungs (Figure 1A,B) and the spleens (Figure 1C) of these mice. There was no difference in the population of naïve CD19^+^IgD^+^IgM^+^ cells in either organ (Figure 1B,C) (similar data was seen at day 60– not shown). These data suggest that within the B cell population in the lungs of infected AID^−/−^µS^−/−^ mice there is a significant increase in both total B cells as well as activated IgD^−^IgM^+^ B cells relative to C57BL/6 mice, these cells likely reflect a population that are activated but unable to class switch and therefore accumulate at this stage of development.

To determine whether mice with increased numbers of B cells were more susceptible to Mtb we compared the growth of bacteria in these mice to C57BL/6 controls. For the first 30 days following aerosol challenge with a moderately high dose of Mtb, C57BL/6 and AID^−/−^µS^−/−^ mice have similar bacterial loads in the lungs and spleen (Figure 1D). Thereafter C57BL/6 mice were able to reduce bacterial burden whereas AID^−/−^µS^−/−^ mice allowed continued, if slowed, bacterial expansion and developed a significantly higher bacterial burden in the lungs and spleens (Figure 1D). AID^−/−^µS^−/−^ mice also showed signs of illness and decreased survival beginning 3 months after infection, whereas C57BL/6 mice remained healthy throughout the experiment (Figure 1E).

AID^−/−^µS^−/−^ mice have several deficiencies associated with B cell function and we wanted to determine whether either of these alone was sufficient to confer similar susceptibility to Mtb. To do this we infected mice lacking one of the genes of interest (i.e. AID^−/−^ or µS^−/−^) and compared bacterial growth to C57BL/6 controls. Importantly, bacterial burdens in the lungs and spleens of AID^−/−^ (Figure 1F) or µS^−/−^ mice (Figure 1G) were comparable to those seen in C57BL/6 mice with µS^−/−^ mice showing a reproducible level of protection in both the lung and spleen at day 20 post infection, this however was lost by day 30. Previous studies have shown increased susceptibility of B cell deficient mice to a similar dose of bacteria as used here. To investigate the role of B cells in our model we also infected B cell deficient mice (µMT) which totally lack B cells. We did not observe any increased susceptibility in the lung (Figure 1 H) relative to C57BL/6 mice but did note lower bacterial burdens in the spleens of these mice during the early stages of infection (Figure 1H), which was consistent with a previous report [21]. These observations indicate that neither the absence of both B cells and antibodies (µMT mice) nor the presence of B cells with some circulating immunoglobulin (µS^−/−^ or AID^−/−^ mice) dramatically impacts protective immunity to Mtb. In our model therefore, the presence of B cells lacking the ability to class switch or secrete antibody (as in AID^−/−^µS^−/−^ mice) is detrimental for the control of Mtb growth.

### B cell Depletion Allows for Improved Control of Mtb in the Spleen of AID^−/−^µS^−/−^ Mice

**Figure 2:**
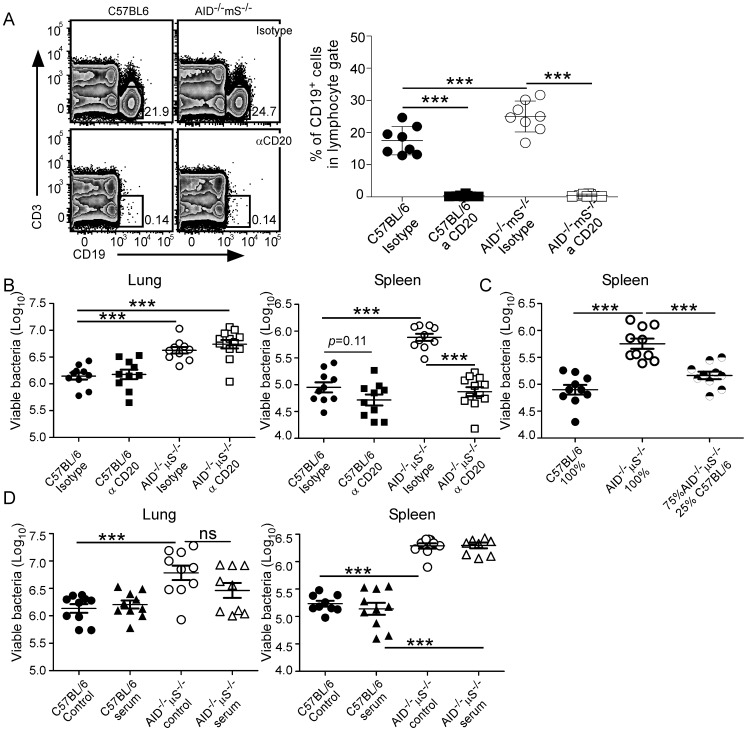
B cell depletion in Mtb-infected AID^−/−^µS^−/−^ mice allows for improved control of bacterial burdens in the spleen, but not in the lung. C57BL/6 (closed symbols) and AID^−/−^µS^−/−^ (open symbols) mice were infected as for Figure 1 and then treated every other week starting either day 0 or day 30 with an isotype control antibody (circles) or with an anti-mouse CD20 antibody (αCD20, squares) and (A) flow cytometry was used to determine depletion of CD19^+^CD3^−^ cells at day 30 (depletion was maintained through day 60 - not shown). (B) The bacterial burden was determined by plating the lung and spleen on agar and counting viable colony forming units. (C) Radiation bone marrow chimeras were made with bone marrow from C57BL/6 (closed circles) AID^−/−^µS^−/−^ (open circles) or a mixture of the two (half filled circles) and then infected as for Figure 1 and the bacterial burden in the spleen measured. (D) C57BL/6 (closed symbols) and AID^−/−^µS^−/−^ (open symbols) mice were infected as for Figure 1 and then treated every three days starting at day 15 with normal mouse serum (triangles) or were left untreated (circles). Bacterial burden was assessed at day 60. Data from two separate experiments showing the same results independently have been combined in A–D (*n* = 8–10 mice per group). **, p<0.01; ***, *p*<0.001 by ANOVA with Tukey’s multiple comparisons post-test.

As AID^−/−^µS^−/−^ mice are more susceptible to Mtb but µMT mice are not, we hypothesized that the stalled B cells identified in the AID^−/−^µS^−/−^ mice (Figure 1A–C) are detrimental to the control of Mtb in the mouse. To test this hypothesis we infected C57BL/6 and AID^−/−^µS^−/−^ mice with a moderately high dose of Mtb and then treated mice with an anti-CD20 antibody to specifically deplete B cells [41], [43], [44]. Since the susceptibility of AID^−/−^µS^−/−^ to Mtb becomes clear only by day 60 post-infection, we started anti-CD20 treatment either at day 0 or day 30 post-infection and determined bacterial loads at day 60 post-infection. The capacity of the anti-CD20 treatment to deplete CD19^+^ B cells was determined by flow cytometry and was found to result in virtual elimination of CD19^+^ cells in the lung (Figure 2A). As the outcome of treatment from 0–60 and 30–60 days was similar, the data were combined (Figure 2B). Depletion of B cells did not significantly impact Mtb bacterial burden in the lungs of C57BL/6 mice but trended toward a slight reduction in the spleens of these mice (Figure 2B). While the treatment did not impact the bacterial load in the lungs of infected AID^−/−^µS^−/−^ mice it did significantly reduce bacterial burden in the spleen of Mtb-infected AID^−/−^µS^−/−^ mice returning them to the levels seen in C57BL/6 mice (Figure 2B). In an alternate approach to address the impact of non hematopoietic cells in the susceptibility of the AID^−/−^µS^−/−^ mice to Mtb and to see whether the detrimental impact of B cells in the spleens of AID^−/−^µS^−/−^ mice could be altered by the presence of intact B cells, we made radiation bone marrow chimeras. In these mice the host was a C57BL/6 mouse which was reconstituted with either100% C57BL/6 or 100% AID^−/−^µS^−/−^ bone marrow or with 25% C57BL/6 combined with 75% AID^−/−^µS^−/−^ bone marrow. As expected the 100% C57BL/6 chimeras controlled bacterial burden in the spleen better than the 100% AID^−/−^µS^−/−^ chimeras (Figure 2C). Importantly, the addition of 25% normal bone marrow to the AID^−/−^µS^−/−^ bone marrow reversed the phenotype to that of the C57BL/6 suggesting that the normal B cells modulated the impact of the altered AID^−/−^µS^−/−^ B cells. Together these data demonstrate that there is a direct negative impact of the AID^−/−^µS^−/−^ B cells in the control of Mtb in the spleens these mice.

While the C57BL/6 B cells could be directly modulating the activity of the AID^−/−^µS^−/−^ B cells, the C57BL/6 B cells could also be producing sufficient immunoglobulin to alter the phenotype of the AID^−/−^µS^−/−^ cells. To address this we measured the level of immunoglobulin in the chimeric mice and found that there was no significant difference between the levels of IgG1, IgG2b and IgM in the 25%/75% mixed chimera relative to the 100% B6 chimera while the 100% AID^−/−^µS^−/−^ chimera totally lacked circulating immunoglobulin (n = 5 Student’s *t* test on log transformed titers of each isotype). To determine if immunoglobulin alone could reverse the susceptibility of AID^−/−^µS^−/−^ mice we delivered normal mouse serum to both C57BL/6 and AID^−/−^µS^−/−^ infected mice and determined the impact on bacterial burden. We found however that while there was a very modest trend towards reduced bacterial burden in the lung, this did not reach statistical significance and was not seen at all in the spleen. These data suggest that circulating immunoglobulin alone is unable to restore control of bacterial growth in the AID^−/−^µS^−/−^ mice.

### Expression of the Type 1 Immune Response is not Affected in AID^−/−^µS^−/−^ Mice

**Figure 3:**
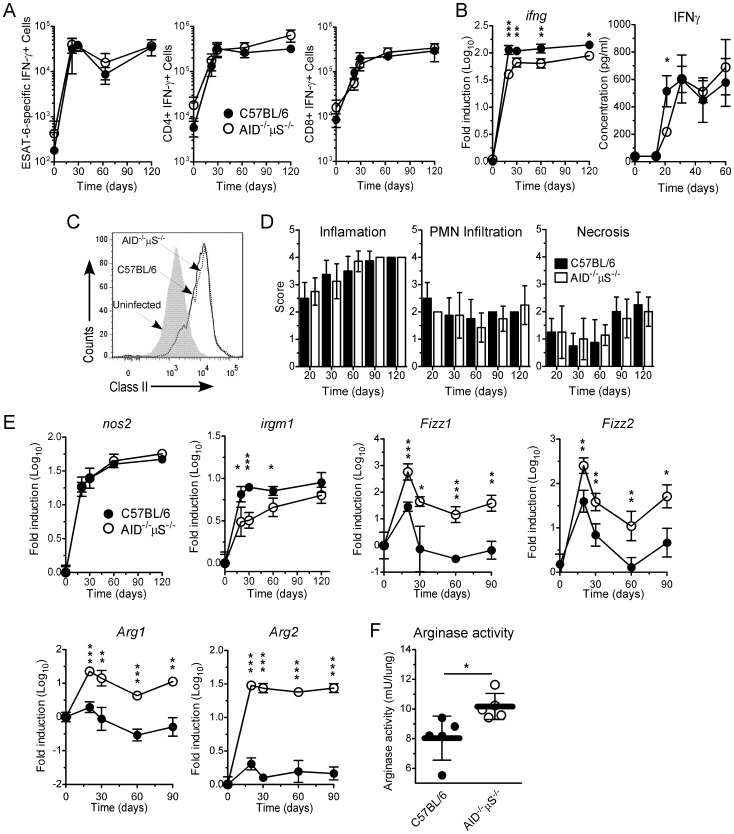
Type 1 immune responses are similar but myeloid cell activation is different between C57BL/6 and AID^−/−^µS^−/−^ Mtb-infected mice. (A–F) C57BL/6 (filled symbols) and AID^−/−^µS^−/−^ (opened symbols) mice were infected as described for Figure 1. (A) Lung cells were cultured with ESAT_1–20_ peptide to determine the number of IFN-γ-producing ESAT_1–20_-specific cells. The number of CD3^+^CD4^+^IFN-γ^+^ or CD3^+^CD8^+^IFN-γ^+^ was determined by flow cytometry. (B) The presence of mRNA for *ifng* in the lungs of infected mice was determined by real-time RT-PCR. (B) IFN-γ protein was determined in lung homogenates by Luminex. (C) MHC-II expression on CD11c+ cells (expressed by alveolar macrophages and dendritic cells) derived from the lungs of uninfected (shaded curve) or infected C57BL/6 (solid line) or AID^−/−^µS^−/−^ mice (dashed line) was determined by flow cytometry. (A–C) One experiment representative of 3 independent experiments is shown. *n* = 4 mice per group. *, p<0.05; **, p<0.01; ***, *p*<0.001 by Student’s *t* test. (D) At specific time points after infection, the caudal lobe of the lung from each mouse was processed for histologic analysis and stained using H&E. Sections were screened and scored for inflammation, PMN infiltration and necrosis by a pathologist in a blinded manner (0: absent; 1: minimal; 2: mild; 3: moderate; 4: marked). One experiment representative of 2 independent experiments is shown. *n* = 4 mice per group. (E) At different time points after infection, RNA was extracted from lung tissue and analyzed by real-time PCR for the expression of *nos2, irgm1, fizz1, fizz2, arg1 and arg2*. (F) At day 60 post-infection, arginase activity was determined in the lungs of C57BL/6 (filled symbols) and AID^−/−^µS^−/−^ (open symbols). One experiment representative of 2 independent experiments is shown. *n* = 4–5 mice per group. *, p<0.05; **, p<0.01; ***, *p*<0.001 by Student’s *t* test.

While the increased susceptibility in the spleen is clearly dependent upon the altered B cell population, the reason for the increased susceptibility in the lung was not clear. To define the cause of the susceptibility in the lung we compared several parameters of immune responsiveness between the lungs of Mtb-infected C57BL/6 and AID^−/−^µS^−/−^ mice. Control of Mtb is dependent upon the activation of antigen-specific CD4^+^ T cells followed by migration of these cells to the lung to activate infected myeloid cells and to coordinate the formation of an organized granuloma [11]. We therefore compared the type 1 response between C57BL/6 and AID^−/−^µS^−/−^ Mtb-infected mice and found that while the number of antigen-specific cytokine producing cells was similar between these organs (Figure 3 A), a small reduction of IFN-γ mRNA and early protein expression (day 20) was detected in the lungs of Mtb-infected AID^−/−^µS^−/−^ mice (Figure 3B). To assess the impact of the lower IFN-γ protein in the lungs in terms of myeloid activation, we compared the expression of MHC class II by lung CD11c+ cells (expressed by both dendritic cells and alveolar macrophages) at day 20 post-infection and found it to be similar in both groups of infected mice by day 20 (Figure 3C). These data suggest that, despite modestly reduced levels of IFN-γ in AID^−/−^µS^−/−^ mice, there is rapid activation of the lung myeloid lineage.

Previous studies had suggested altered inflammatory responses in B cell deficient mice and so an extensive histological examination of lung samples was performed by a veterinary pathologist and no major differences in the degree of inflammation, polymorphonuclear cell infiltration or necrosis (Figure 3D) were found throughout infection. These data suggest that the increased susceptibility to Mtb in the lungs of the AID^−/−^µS^−/−^ is not associated with altered expression of type 1 immune responses nor with any overt pathologic consequences.

While expression of MHC class II is a sign that phagocytes in the lung are activated, it does not directly address their ability to limit bacterial growth. Classical macrophage activation is critical for controlling Mtb infection through IFN-γ-induced expression of effector genes such as *nos2*. We therefore compared the potential function of the phagocytes within the lungs of the C57BL/6 and AID^−/−^µS^−/−^ mice using gene expression. We measured the induction of two antimycobacterial genes, that for the inducible nitric oxide synthase (iNOS) (*nos2*) [45] and that for the immunity-related GTPase family M member 1 gene (*irgm1*) [46] and found that while there was a similar expression of *nos2* in both groups of Mtb-infected mice, there was reduced expression of *irgm1* by AID^−/−^µS^−/−^ mice at early stages of infection (Figure 3E), consistent with a reduced expression of IFN-γ at this same time points (Figure 3B). While expression of enzymes such as *nos2* reflects the ability of the phagocytes to express toxic nitrogen radicals, this function is also dependent on the availability of substrate for iNOS. Concomitant expression of arginase I which hydrolyzes L-arginine into urea and L-ornithine, can limit availability of L-arginine as a substrate for nitric oxide production by iNOS [47], [48], [49]. Thus, while both C57BL/6 and AID^−/−^µS^−/−^ Mtb-infected mice express similar levels of *nos2*, we hypothesized that the activity of iNOS could be impacted by increased expression of arginases in AID^−/−^µS^−/−^ mice. Arginase genes are expressed alongside other genes such as *fizz1* and *fizz2* both of which are significantly upregulated in the lungs of the Mtb-infected AID^−/−^µS^−/−^ mice compared to infected C57BL/6 mice (Figure 3E). Indeed, we found that while there was a transient increase in both arginase I and arginase II (Figure 3E) in the lungs of C57BL/6, there was sustained and higher expression of both arginase I and arginase II in the lungs of Mtb-infected AID^−/−^µS^−/−^ mice (Figure 3E). We also found increased arginase activity in the lungs of AID^−/−^µS^−/−^ mice at day 60 post-infection (Figure 3F). These data suggest that the AID^−/−^µS^−/−^ mice have a lung environment that is different from the C57BL/6 mice in that there are higher levels of genes associated with alternatively activated macrophages at time points even prior to differences in bacterial burden.

### IL-10 Signaling Blockade Reverses the Susceptibility of AID^−/−^µS^−/−^ Mice to Mtb

**Figure 4:**
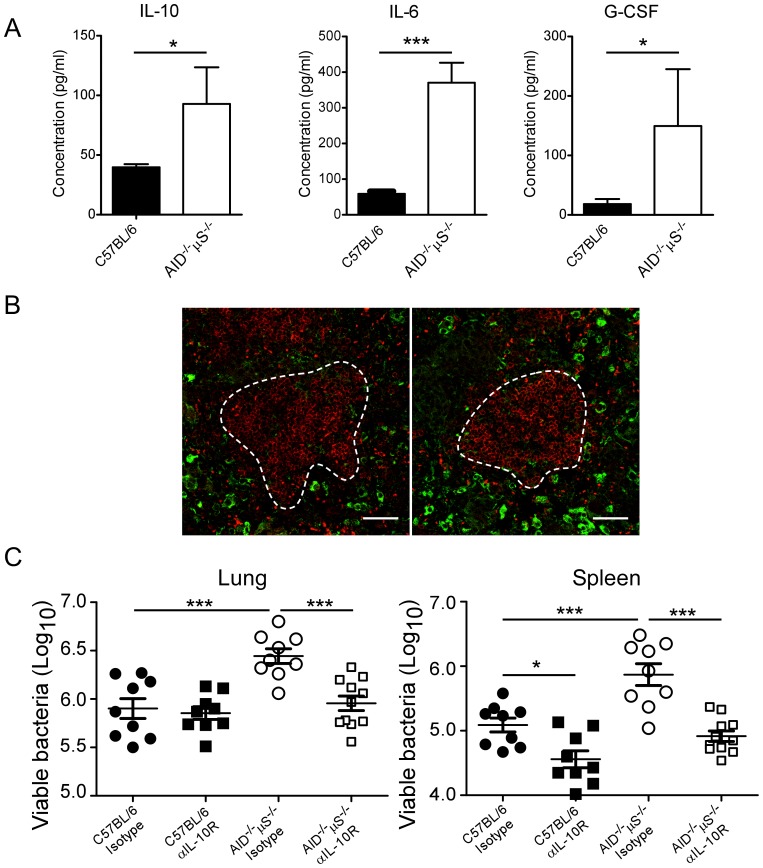
Blocking of IL-10R improves the ability of AID^−/−^µS^−/−^ mice to control Mtb. C57BL/6 and AID^−/−^µS*^−/−^* mice were infected as described for Figure 1. (A) The concentration of IL-10, IL-6 and G-CSF in lung homogenates of Mtb-infected mice was determined by Luminex. One experiment representative of 2 independent experiments is shown (*n* = 4). *, p<0.05; **, p<0.01 ***, *p*<0.001 by Student’s *t* test. (B) Lung sections from mice infected for 60 days as in Figure 1 were stained for B220 (red) and IL-10 (green), white dashed line represents edge of B cell follicle area (bar represents 50 microns). Representative sections shown for C57BL/6 (left) and AID^−/−^µS^−/−^ (right panel), reproduced in five other mice and in 2 separate experiments. (C) C57BL/6 (filled symbols) and AID^−/−^µS^−/−^ (open symbols) mice were infected as described in Figure 1. At day 0 and once a week thereafter, mice were treated with anti-IL-10R antibody (squares) or with the same concentration of an isotype control antibody (circles). Lung and spleen bacterial burden was determined at day 60 post-infection. One experiment representative of two independent experiments is shown (*n* = 5). *, p<0.05; ***, *p*<0.001 by one-way ANOVA with Tukey’s multiple comparisons post-test.

Arginase expression during intracellular infections has been shown to be dependent on STAT-3 activation and the cytokines IL-6, G-CSF and IL-10 [47]. We therefore measured these cytokines in the lung homogenates during Mtb infection and found increased IL-10, IL-6 and G-CSF (Figure 4A). IL-10 in particular is likely to reduce the efficacy of the phagocytes in the lung and so we determined the source of the IL-10 within the lung by immunohistological staining. Mtb-infected C57BL/6 and AID^−/−^µS^−/−^ lung (Figure 4B) and spleen sections (not depicted) were positive for IL-10 (depicted in green) and B cells (B220, depicted in red). However, we found no substantial B220^+^ IL-10^+^ cells in the lung, rather the IL-10 positive cells were located outside the B cell follicles (delineated by the white lines) and had a macrophage-like appearance (Figure 4B), suggesting that the B cells were not the source of excess IL-10.

To determine whether the increased IL-10 observed in the lungs of the Mtb-infected AID^−/−^µS^−/−^ mice was responsible for the increased susceptibility seen in this organ, we treated both C57BL/6 and AID^−/−^µS^−/−^ mice with anti-IL-10R antibody and compared bacterial number at day 60. We found that while anti-IL-10R blockade did not impact bacterial burdens in the lungs of C57BL/6 mice, it reduced the bacterial burden in the spleens of these mice (Figure 4C). Importantly, the addition of anti-IL-10R blockade to the Mtb-infected AID^−/−^µS^−/−^ mice resulted in the reversion of the susceptibility phenotype in both the lungs and spleen (Figure 4C) to that seen in C57BL/6 mice. Taken together, these data demonstrate that the enhanced susceptibility of AID^−/−^µS^−/−^ mice to Mtb in the lung is related to altered macrophage activation and is dependent upon high levels of IL-10 seen in the lungs of these mice.

## Discussion

The presence of activated B cells and high levels of Mtb-specific antibody during tuberculosis prompted us to investigate the relative impact of B cells in the absence of immunoglobulin in the protective immune response to TB. We found that B cells can be directly detrimental to control of Mtb in the spleen but that excess IL-10 is directly responsible for the increased susceptibility seen in mice that have B cells but lack circulating immunoglobulin. Delivery of exogenous immunoglobulin alone failed to restore the capacity of the mutant mice to control bacterial growth in the lung or spleen. We found that macrophages are the main cellular source of IL-10 during infection and that the macrophages in these mice produce arginase as well as iNOS and are likely less efficient at controlling bacterial growth. We also show that there is no apparent role for immunoglobulin in the initiation and expression of type 1 immune responses specifically the generation of antigen-specific Th1 cells.

An intriguing aspect to our studies is that the results for the spleen and lung were quite different. It was clear that the B cells present in the AID^−/−^µS^−/−^ mice were detrimental to control in the spleen but that these B cells could be controlled by B cells from normal mice. There was a slight trend toward reduced bacterial burden in the C57BL/6 mice depleted of B cells (Figure 2B) as well as increased control in the spleens of B cell deficient mice (Figure 1H) [21]. Together these data support a role for B cells acting at the level of the spleen in Mtb-infected mice. As we found that B cell populations in the C57BL/6 and AID^−/−^µS^−/−^ mice differed at the level of CD19^+^IgD^−^IgM^+^ cells it is possible that within this subset of B cells there are cells which impact bacterial control in the spleen but not in the lung. Further characterization of this population is therefore warranted.

The impact of IL-10 in the protective immune response to TB in the mouse model has been extensively covered in the literature [48], [50], [51], [52], [53]. The current understanding in the field is that while IL-10 does not compromise the initiation of the T cell response it reduces its efficacy by inducing arginase expression in activated macrophages, which in turn reduces the available L-arginine for nitric oxide production [47], [48], [49]. Our data support this current understanding, as the excess IL-10 seen in the AID^−/−^µS^−/−^ mice has limited impact on the T cell response and the classical aspects of macrophage activation but is associated with elevated expression of arginase genes and arginase activity, thereby limiting the ability of the macrophages to control bacterial growth. Our observation of increased IL-10, IL-6 and G-CSF in the infected AID^−/−^µS^−/−^ mice may reflect increased induction of these cytokines by the mycobacteria via an MyD88-dependent pathway as reported previously [47]. Why there is an increase in this signaling in the AID^−/−^µS^−/−^ lungs is unclear at this point but importantly, it precedes the differences in bacterial number seen in the lung. Regardless of the reason for increased production, it is likely that these cytokines promote expression of arginase 1 in an autocrine and paracrine manner [47]. As we also see a modest upregulation of *fizz1* and *fizz2*, it is possible that IL-4, even in small amounts may be influencing the macrophages in the AID^−/−^µS^−/−^ mice [47] as IL-10 signaling can induce the upregulation of IL-4Rα [54]. The source of the excess IL-10 in the current model is of interest as B cells have been shown to produce IL-10 [55]. However our immunohistochemical data (along with PCR on CD19 cells from the lung - data not shown) suggest that B cells are not the primary source of IL-10 in our model.

It was previously shown that lung cells isolated from Mtb-infected mice that have had the stimulatory FcγR chain deleted produce higher levels of IL-10 upon ex vivo re-stimulation, suggesting that FcγR ligation by circulating immunoglobulin may be a key mechanism in the regulation of IL-10 production during Mtb infection [30]. However, our failure to reverse susceptibility by delivery of serum weakens the possibility that circulating immunoglobulin is regulating the activating state of macrophages in our model. It is possible that we failed to deliver the immunoglobulin to the correct location or may not have delivered the right isotypes in the correct proportions. While immunoglobulin alone may not be able to moderate the susceptibility of the AID^−/−^µS^−/−^ mice it is possible that specific immunoglobulin may play a role. However when one considers the lack of susceptibility in the AID single mutant (Figure 1F) a role for specific antibody is not clear. These mice lack the ability to class switch or somatically hypermutate [37] and therefore the circulating immunoglobulin in these mice, even if it has some specificity, will be similar to the IgM in the naïve serum. Thus while our data do not disprove a role for circulating immunoglobulin they do not support a role for immunoglobulin in regulating the immune response to Mtb.

In our study, µMT mice (which lack both circulating immunoglobulin and B cells) are not more susceptible to Mtb. This further suggests that the simple lack of circulating immunoglobulin is not sufficient to promote the macrophage phenotype seen in the infected AID^−/−^µS^−/−^ mice. In other studies however, µMT mice are more susceptible to Mtb and lung cells isolated from these mice produced higher levels of IL-10 when compared to C57BL/6 mice [23]. This apparent conflict between data sets from seemingly similar experiments may reflect differences in immune status of the mice in different facilities. Indeed, B cells and immunoglobulin, specifically IgA, have been shown to be critical for maintaining gut microbial homeostasis which, in turn, plays an important role in the regulation of immune responses in the lung [27]. We attempted to address the role of gut microbiota in the susceptibility of the AID^−/−^µS^−/−^ mice, by continuing to feed both AID^−/−^µS^−/−^ and C57BL/6 mice with antibiotic treated food (Smx-Tmp) throughout infection. We found that both C57BL/6 and AID^−/−^µS^−/−^ antibiotic treated mice were as susceptible to Mtb as were non-treated mice (data not shown), suggesting that gut microbial homeostasis is not the critical element in the susceptibility of AID^−/−^µS^−/−^ mice to Mtb.

Understanding the impact of B cells in mycobacterial disease is important as B cell depletion therapies are becoming an option in the treatment of autoimmune and chronic inflammatory diseases [56], [57]. Further, with the increased use of anti-B cell therapies in areas of the world where TB is a more significant risk than in the US and Europe it is critical that the incidence of mycobacterial disease in patients receiving such therapies be monitored and reported. Our data suggest that anti-B cell treatment may actually reduce the likelihood of disseminated or extra-pulmonary disease. These outcomes are however difficult to predict. Further, increasing our understanding of how the protective immune response is impacted by B cells will allow us to better manipulate these cells during TB.
